# Frequency of Th17 CD4+ T Cells in Early Rheumatoid Arthritis: A Marker of Anti-CCP Seropositivity

**DOI:** 10.1371/journal.pone.0042189

**Published:** 2012-08-03

**Authors:** Irene Arroyo-Villa, María-Belén Bautista-Caro, Alejandro Balsa, Pilar Aguado-Acín, Laura Nuño, María-Gema Bonilla-Hernán, Amaya Puig-Kröger, Emilio Martín-Mola, María-Eugenia Miranda-Carús

**Affiliations:** 1 Department of Rheumatology, Hospital Universitario La Paz-IdiPaz, Madrid, Spain; 2 Laboratorio de Inmuno-Oncología, Hospital General Universitario Gregorio Marañón, Madrid, Spain; University Hospital Jena, Germany

## Abstract

**Objective:**

To examine the frequency and phenotype of Th17 cells in the peripheral blood of early RA (eRA) patients.

**Methods:**

CD4+ T cells were isolated from the peripheral blood of 33 eRA patients, 20 established RA patients and 53 healthy controls (HC), and from the synovial fluid of 20 established RA patients (RASF), by ficoll-hypaque gradient and magnetical negative selection. After polyclonal stimulation, the frequency of Th17 and Th1 cells was determined by flow cytometry and concentrations of IL-17, IFN-γ, TNF-α and IL-10 were measured by ELISA in cell-free supernatants.

**Results:**

When all of our eRA patients were analyzed together, a significantly lower percentage of circulating Th17 cells and a lower CD4-derived IL-17 secretion were observed in comparison with HC. However, after stratifying by anti-CCP antibody status, circulating Th17 cells were decreased in anti-CCP(+) but not in anti-CCP(-)-eRA. All Th17 cells were CD45RO+CD45RA- and CCR6+. Dual Th17/Th1 cells were also exclusively decreased in anti-CCP(+)-eRA. Circulating Th17 and Th17/Th1 cells were negatively correlated with anti-CCP titres. When anti-CCP(+)-eRA patients were retested one year after initiating treatment with oral methotrexate, their circulating Th17 frequency was no longer different from HC. Of note, the percentage of circulating Th1 cells and the secretion of CD4-derived IFN-γ, TNF-α and IL-10 were not different between eRA patients and HC. In established RA patients, circulating Th17 and T17/Th1 cell frequencies were comparable to HC. In RASF, both Th17 and Th1 cells were increased when compared with blood of eRA patients, established RA patients and HC.

**Conclusion:**

Decreased circulating Th17 levels in eRA seem to be a marker of anti-CCP seropositivity, and return to levels observed in healthy controls after treatment with methotrexate.

## Introduction

RA, a systemic autoimmune disease, is characterized by chronic joint inflammation, cartilage destruction and bone erosions. Numerous experimental data indicate that IL-17A (henceforth referred to as IL-17) plays an important role in the pathogenesis of RA [1]. IL-17-deficient mice demonstrate a markedly attenuated form of collagen-induced arthritis (CIA) [2], neutralization of IL-17 during induction of experimental arthritis suppresses the onset of disease [3], and anti-IL-17 therapy in established CIA is associated with a significant reduction of severity [4]. IL-17 is implicated in the development of bone erosions by altering the RANKL/OPG balance [5], and its action may be independent of TNF-α [6], [7]. In human studies, IL-17 is spontaneously produced by RA synovial membrane cultures [8], high levels have been observed in the synovial fluid of patients with RA [8], [9], IL-17 producing CD4+ T cells have been detected in RA synovial membranes [10]–[12] and neutralization of IL-17 seems to be effective in RA clinical trials [13]. Several sources of IL-17 have been described: Th-17 cells, which are a subset of CD4+ helper T cells, mast cells, NK cells and γδ T cells, and all of them may contribute to the pathogenesis of inflammatory arthritis [14]–[17]. An altered percentage of Th17 cells has been described in the peripheral blood and synovial fluid of RA patients, but to date conflicting data have been reported [18]–[25].

Our objective was to examine the frequency and phenotype of Th17 cells in the peripheral blood of early RA patients, and in the synovial fluid of patients with established RA. We were also interested in determining which cell type is the main producer of IL-17 in eRA peripheral blood and RA synovial fluid. Our early arthritis clinic allowed the study of T cells from early RA patients who have not received disease modifying drugs (DMARDs) or steroids, thereby minimizing interference of drugs with ex vivo T cell responses.

## Patients and Methods

### Ethics Statement

The study was approved by the Hospital La Paz - IdiPAZ Ethics Committee, and all subjects provided written informed consent.

### Patients

Peripheral blood was obtained from 33 early RA patients and from 33 age and sex-matched healthy controls. Early RA patients fulfilled at least four 1987 American College of Rheumatology criteria [26], had never received disease-modifying drugs or corticosteroids, and had a disease duration of less than 6 months. La Paz University Hospital in Madrid, Spain, has a monographic clinic that takes care of early arthritis patients referred from a wide primary care area, and this facilitated recruitment of untreated early RA patients for the present study. Among early RA patients there were 3 male and 30 female, 17 (52%) tested positive for IgM rheumatoid factor, and 15 (45%) tested positive for anti-CCP antibodies; their age was 53.4+13.1 years (mean + SD), and disease activity score 28 (DAS28) [27] at first evaluation was 4.8+1.4 (mean + SD).

Eight anti-CCP positive eRA patients donated blood for a second time, one year after the initiation of treatment with oral MTX and low-dose prednisone. At the time of the second blood drawing, prednisone had been discontinued in all except for 2 patients, who were taking 2.5 mg dayly, and all of them were taking oral MTX at doses of 10 to 25 mg. These patients were 1 male and 7 female, aged 51.63±15.17 years (mean ± SD). 4 out of these 8 subjects had achieved remission as defined by a DAS28 score below 2.6 [28]. The remaining four patients had experienced a significant clinical improvement, with a decrease in the DAS28 of >2.0 points, but they still demonstrated significant disease activity associated with a DAS28>2.6. Blood was also obtained from the 8 healthy subjects who had previously acted as controls for these patients.

Synovial fluid (SF) was obtained from the knee joints of 20 patients with established RA who were receiving treatment with oral methotrexate (MTX) and low-dose prednisone; 13 of these patients (65%) tested positive for IgM rheumatoid factor and 11 (55%) tested positive for anti-CCP antibodies. In addition, peripheral blood was obtained from 20 patients with established RA who were receiving treatment with oral methotrexate (MTX) and low-dose prednisone, and from 20 age- and sex- matched controls. 11 of these patients (55%) tested positive for IgM rheumatoid factor and 11 (55%) tested positive for anti-CCP antibodies.

### Isolation of CD4+ T Cells

Mononuclear cells were isolated from human blood and synovial fluid by density centrifugation over Ficoll-Paque Plus (GE Healthcare, Chalfont St. Giles, United Kingdom). CD4+ T cells were subsequently purified by negative selection in an Automacs (Miltenyi Biotec, Bergisch Gladbach, Germany) using the “CD4+ T Cell Isolation Kit II” from Miltenyi Biotec, containing a cocktail of biotin-conjugated antibodies against CD8, CD14, CD16, CD19, CD36, CD56, CD123, γ/δ TCR and Glycophorin A, followed by anti-Biotin MicroBeads. Isolated CD4+ T cells were 98% pure and free of detectable CD14+ monocytes, CD8+ T cells, CD56+ NK cells, CD19+ B cells, and γδ T cells.

T Cell StimulationImmediately after isolation, CD4+ T cells were cultured and stimulated in 24-well plates (10^6^ cells/well) containing RPMI 1640 medium (Lonza) with 10% FCS, 2 mM L-glutamine, 50 U/ml penicillin, 50 µg/ml streptomycin and 50 µM 2-mercapto-ethanol (“complete RPMI medium”). Two different activation strategies were undertaken. T cells were stimulated for 16 h with phorbol myristate acetate (PMA) (10 nM) and ionomycin (2 µM), in the presence or absence of 4 µM monensin (all three from Sigma-Aldrich). In addition, cells were cultured for 1–4 days in the presence of a soluble anti-CD3 IgE subclass mAb (T3/4.E, Sanquin, Amsterdam, The Netherlands, formerly CLB) (0.5 µg/ml) plus anti-CD28 (1 µg/ml) (BD Pharmingen) and anti-CD49d mAbs (1 µg/ml) (BD Pharmingen), and restimulated for the last 6 hours with PMA and ionomycin, with or without monensin.

### Intracellular Cytokine Staining, Surface Staining, and Flow Cytometry

Fluorochrome-conjugated mAbs from BD Pharmingen were used to examine the expression of the phenotypical markers CD3, CD4, CD8, CCR6, CCR4, CD45RO and CD45RA. Surface IL-23R was detected with a biotinylated goat anti-human IL-23R antibody (R&D Systems), followed by a FITC labeled avidin (BD Pharmingen). For intracellular cytokine staining, T cells were washed with PBS/2% FCS/0.01% NaN3, permeabilized for 10 min with FACS permeabilizing solution 2 (BD Pharmingen), washed again, and incubated on ice for 1 h with an APC-labelled anti-IFN-γ mAb (clone B27; BD Pharmingen), a phycoerythrin (PE)-labelled anti-IL17A mAb (clone eBio64DEC17; eBioscience), a FITC-labeled anti-TNF-α mAb (clone Mab11, BD Pharmingen), a PE-labeled anti-IL10 mAb (clone B-T10, Miltenyi Biotech) or fluorochrome-labeled isotype control mAbs. After washing once with PBS/2% FCS/0.01% NaN3 and once with PBS, cells were resuspended in 1% paraformaldehyde and analyzed in a FACSCalibur flow cytometer using CellQuest software (BD Biosciences).

### ELISAs

Cell-free culture supernatants were collected and stored at -80°C. ELISAs for IL-17A were performed using a kit from eBioscience. ELISAs for TNF-α, IL-10, and IFN-γ were performed using kits from BD Biosciences following the manufacturer’s instructions.

### Statistical Analysis

Comparison between groups was by Mann-Whitney test. Paired samples were compared using a Wilcoxon matched pairs signed rank sum test. When appropriate, Bonferroni correction for multiple comparisons was applied. Correlations were analyzed using Spearman’s rank correlation coefficients. All analyses were performed using Prism version 5.0 software (GraphPad Software).

## Results

### Expression of IL-17 by RA CD4+ T Cells

**Figure 1:**
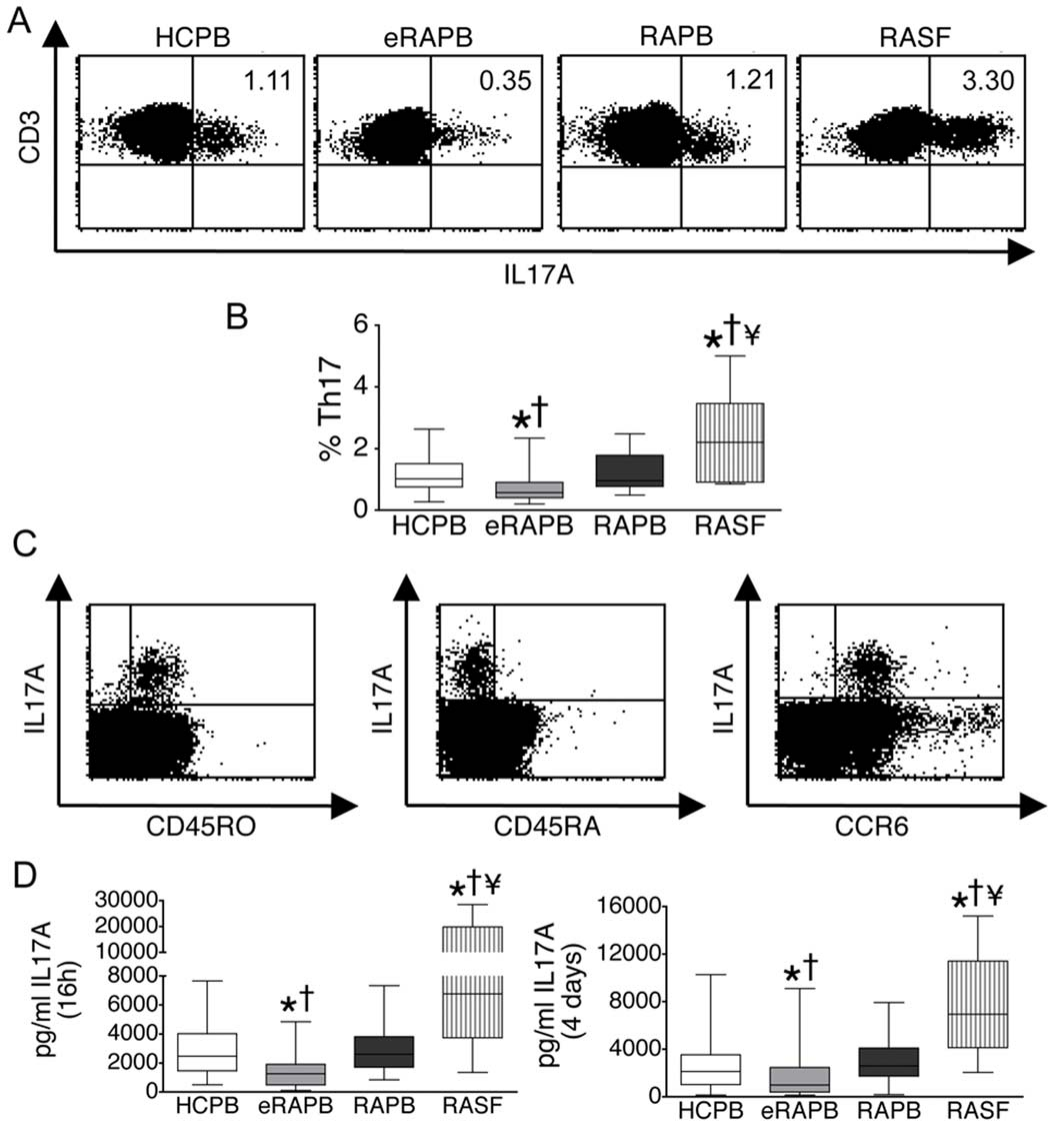
Expression of IL-17A by RA CD4+ T cells. CD4+ T cells were isolated from the peripheral blood of healthy controls (HCPB) (n = 53), the peripheral blood of early RA patients (eRAPB) (n = 33), the peripheral blood of established RA patients (RAPB) (n = 20) and the synovial fluid of established RA patients (RASF) (n = 20), and stimulated with PMA/Ionomycin for 16 h or with anti-CD3/CD28/CD49d for 4 days. A, B. Percentage of CD4+ T cells expressing IL-17A (Th17 cells) after a 16 h stimulation, as determined by flow cytometry. Because the CD4 molecule is downregulated upon stimulation with PMA, shown is CD3 expression on isolated CD4+ T cells. C. Representative flow cytometry dot-plots showing expression of CD45RO, CD45RA and CCR6 versus IL-17A in isolated CD4+ T cells. D. Secretion of IL-17A to the culture medium of CD4+ T cells stimulated for 16 h with PMA/ionomycin or for 4 days with anti-CD3/CD28/CD49d. Box-plots represent the median and interquartile range of all studied subjects, whiskers represent the maximum and minimum values. *p<0.05 vs HCPB; † p<0.05 vs RAPB; ¥ p<0.05 vs eRAPB.

Our working hypothesis stated that the frequency of circulating Th17 cells in eRA would be increased. To our surprise, the frequency of circulating Th17 cells was significantly decreased among eRA patients (0.57%, 0.41–0.91%) (median, interquartile range) in comparison with healthy controls (1.02%, 0.76–1.51%) (Fig. 1A, B). At the same time, the percentage of Th17 cells in the peripheral blood of patients with established RA (0.96%, 0.77–1, 79%) was not different from controls. In contrast, an augmented frequency of Th17 cells was found in the synovial fluid of established RA patients (2.20%, 0.91–3.46%) when compared with circulating frequencies in controls, in eRA and in established RA patients (Fig. 1A, B). All of the Th17 cells expressed the memory phenotypical marker CD45RO, were negative for CD45RA and positive for CCR6 expression (Fig. 1C). The expression of IL-23R and of CCR4 could not be analyzed together with IL-17 since these two molecules are downregulated upon stimulation with PMA/ionomycin. Importantly, the proportion of circulating total CD4+ T cells was not different between eRA and control subjects.

In parallel, the concentration of IL-17 detected by ELISA in culture supernatants of stimulated eRA CD4+ T cells (1247, 472–1911 pg/ml) was significantly decreased when compared with healthy controls (2453, 1455–4012 pg/ml) (Fig. 1D), and this was apparent not only when cells were stimulated overnight with PMA/ionomycin (see above data) but also after stimulation for 4 days with anti-CD3/CD28/CD49d (976, 383–2451 pg/ml for eRA vs 2130, 1019–3525 pg/ml for controls) (Fig. 1D). At the same time, the secretion of IL-17 from peripheral blood CD4+ T cells of established RA patients was not different from controls (2594, 1693–3813 pg/ml after a 16 h stimulation and 2593, 1734–4088 pg/ml after a 4-day stimulation). In contrast, CD4+ T cells from the synovial fluid of established RA patients showed an augmented secretion of IL-17 to the medium (6757, 3724–19800 pg/ml after a 16 h stimulation and 6933, 4128–11420 pg/ml after a 4-day stimulation), when compared with CD4+ T cells from the peripheral blood of eRA patients, established RA patients and controls (Fig. 1D).

Because several reports describe the production of IL-17 by other cell populations (14–17), we additionally isolated γδ T cells, CD8 T cells and NK cells from patients and controls. We could not detect any IL-17 by cytometry or ELISA in these cell subsets from the synovial fluid of RA or from the peripheral blood of eRA, established RA or healthy subjects. Moreover, after thorough depletion of CD4+ T cells from PBMCs, no IL-17 could be detected in culture supernatants after short or long-term stimulation periods, in agreement with work published by Shen et al [29].

### Expression of IFN-γ, TNF-α and IL-10 by RA CD4+ T Cells

**Figure 2:**
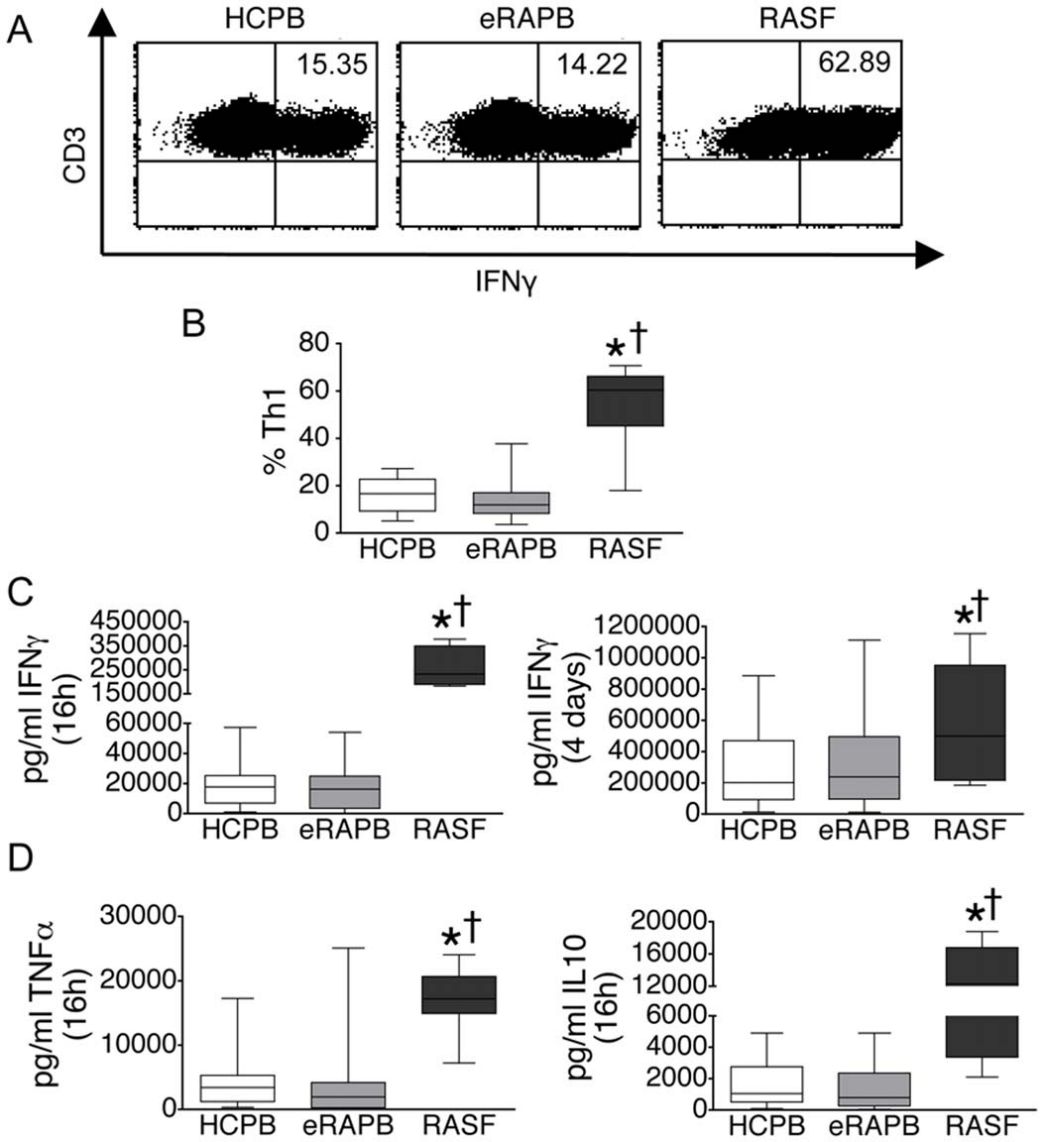
Expression of IFN-γ, TNF-α and IL-10 by RA CD4+ T cells. CD4+ T cells were isolated from the peripheral blood of healthy controls (HCPB) (n = 33), the peripheral blood of early RA patients (eRAPB) (n = 33) and the synovial fluid of established RA patients (RASF) (n = 20), and stimulated with PMA/Ionomycin for 16 h or with anti-CD3/CD28/CD49d for 4 days. A, B. Percentage of CD4+ T cells expressing IFN-γ (Th1 cells) after a 16 h stimulation, as determined by flow cytometry. Because the CD4 molecule is downregulated upon stimulation with PMA, shown is CD3 expression on CD4+ T cells. C. Secretion of IFN-γ by CD4+ T cells stimulated for 16 h with PMA/ionomycin or for 4 days with anti-CD3/CD28/CD49d. D. Secretion of TNF-α and of IL-10 by CD4+ T cells stimulated for 16 h with PMA/ionomycin. Box-plots represent the median and interquartile range of all studied subjects, whiskers represent the maximum and minimum values. *p<0.05 vs HCPB; † p<0.05 vs eRAPB.

Interestingly, the frequency of circulating CD4+ T cells producing IFN-γ (Th1) was comparable between eRA (11.88, 8.22–17.50%) (median, interquartile range) and control subjects (16.58, 9.25–22.64%) (Fig. 2A, B). In addition, no differences were observed between these two groups when determining IFN-γ, TNF-α and IL-10 in supernatants of stimulated CD4 T cells (Fig. 2C, D). In contrast, when compared with CD4+ T cells from the peripheral blood of controls or eRA, augmented frequencies of Th1 cells together with an augmented secretion of CD4-derived IFN-γ, TNF-α or IL-10 were observed in the synovial fluid of established RA patients (Fig. 2, A-D).

### Frequency of Th17/Th1 Cells in RA

**Figure 3:**
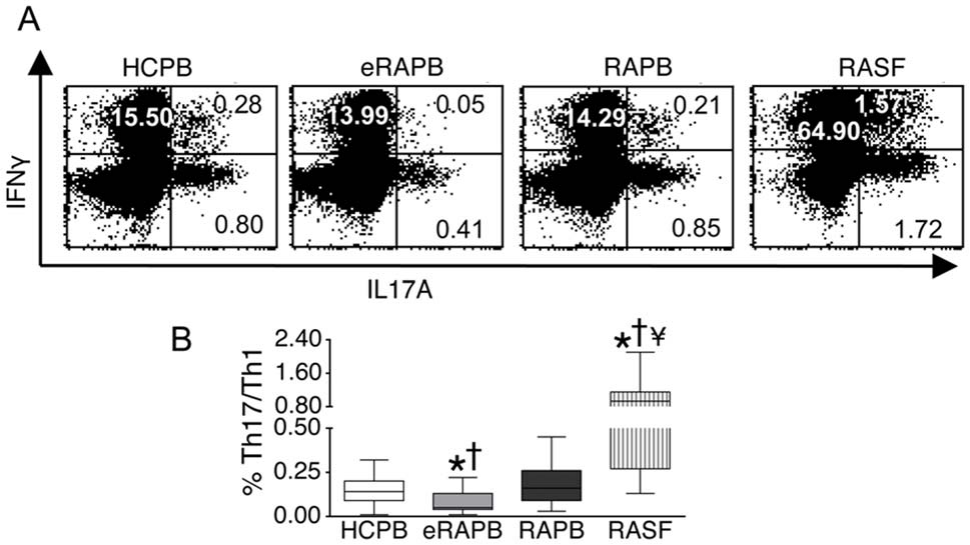
Proportion of Th17/Th1 cells in RA. CD4+ T cells were isolated from the peripheral blood of healthy controls (HCPB) (n = 53), the peripheral blood of early RA patients (eRAPB) (n = 33), the peripheral blood of established RA patients (RAPB) (n = 20) and the synovial fluid of established RA patients (RASF) (n = 20). A, B. Percentage of CD4+ T cells expressing both IL-17A and IFN-γ (Th17/Th1 cells) after a 16 h stimulation with PMA/ionomycin, as determined by cytometry. Box-plots represent the median and interquartile range of all studied subjects, whiskers represent the maximum and minimum values. *p<0.05 vs HCPB; † p<0.05 vs RAPB; ¥ p<0.05 vs eRAPB.

The percentage of cells simultaneously producing IL-17 and IFN-γ (Th17/Th1 cells) [30], was significantly lower in eRA patients (0.05, 0.04–0.13%) when compared with healthy controls (0.14, 0.09–0.20%) (median, interquartile range) (Fig. 3A, B). In contrast, the circulating Th17/Th1 frequency in patients with established RA (0.16, 0.09–0.26%) was not different from controls. At the same time, the percentage of Th17/Th1 cells was higher in RA synovial fluid (0.93, 0.27–1.15%) when compared with the peripheral blood of controls, of eRA patients and of established RA patients (Fig 3A, B).

### Relation of Circulating Th17 Cells with Clinical and Analytical Parameters in eRA

**Figure 4:**
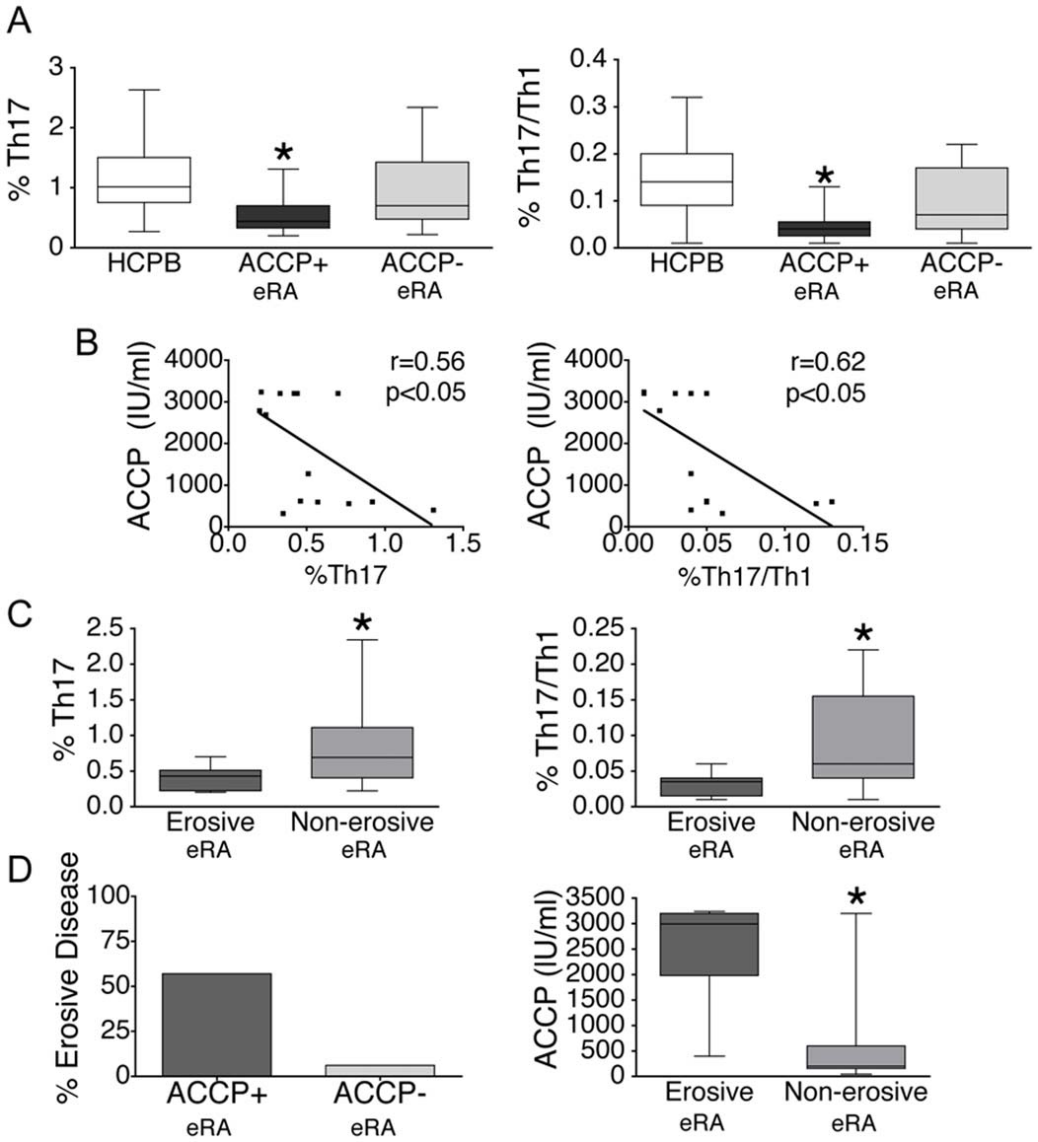
Relation of Th17 and Th17/Th1 frequencies with anti-CCP antibodies and with erosions in eRA. A. Percentage of Th17 and Th17/Th1 cells among CD4+ T cells isolated from the peripheral blood of healthy controls (HCPB) (n = 53), the peripheral blood of anti-CCP+ eRA patients (ACCP+) (n = 15) and the peripheral blood of anti-CCP- eRA patients (ACCP-) (n = 18). *p<0.05 vs HCPB. B. Linear correlation between the percentage of circulating Th17 or Th17/Th1 cells and the anti-CCP antibody titre in anti-CCP+ eRA patients. C. Frequencies of Th17 and Th17/Th1 cells in the peripheral blood of eRA patients with erosive (n = 9) or non-erosive disease (n = 24) at initial presentation. *p<0.05 vs patients with erosive disease. D. Left panel: Percentage of patients with erosive disease at initial presentation among anti-CCP+ and anti-CCP- eRA subjects; Right panel: anti-CCP antibody titres in anti-CCP+ eRA patients with erosive or non-erosive disease at initial presentation. *p<0.05 vs patients with erosive disease. Box-plots represent the median and interquartile range of all studied subjects, whiskers represent the maximum and minimum values.

We then decided to analyze the relation of the circulating Th17 and Th17/Th1 frequencies with clinical and analytical parameters in early RA. The most striking observation came after subdividing eRA patients into two groups based on the anti-CCP antibody status. Anti-CCP positive eRA patients had a significantly lower frequency of Th17 (0.44, 0.33–0.70%) and of Th17/Th1 cells (0.04, 0.03–0.05%) in their peripheral blood when compared with healthy controls (as noted above, Th17: 1.02%, 0.76–1.51% and Th17/Th1 0.14, 0.09–0.20%) (Fig. 4A). Remarkably, the percentage of circulating Th17 (0.70, 0.48–1.43%) and of Th17/Th1 (0.07, 0.04–0.18%) cells in anti-CCP negative eRA patients was not statistically different from controls (Fig. 4A). Of note, in established RA, the circulating Th17 and Th17/Th1 frequencies were not different from controls, for both anti-CCP+ (Th17 0.95, 0.59–1,69%, Th17/Th1 0.15, 0.08–0.29) and anti-CCP- (Th17 1.31, 0.77–1.99, Th17/Th1 0.17, 0.1–0.28) patients.

Interestingly, among anti-CCP+ eRA patients, a significantly negative correlation was observed between the titre of anti-CCP antibodies and the frequency of circulating Th17 cells, and also between the titre of anti-CCP antibodies and the frequency of circulating Th17/Th1 cells (Fig. 4B). No such correlation was observed among anti-CCP+ patients with established RA.

When dividing eRA subjects according to the presence or absence of basal bone erosions in X-rays of the hands and feet, a significantly lower frequency of circulating Th17 and Th17/Th1 cells was observed in patients with erosive (Th17 0.43, 0.22–0.51%; Th17/Th1 0.03, 0.01–0.04%) versus patients with non-erosive disease (Th17 0.69, 0.41–1.02%; Th17/Th1 0.06, 0.04–0.16%) (Fig. 4C). However, after adjusting for anti-CCP antibody status it was evident that this was linked to the strong relation between anti-CCP antibodies and erosions [31]. In fact, the percentage of patients presenting with erosive disease was much higher in anti-CCP+ (57%) versus anti-CCP- eRA (6%) (Fig. 3D); in addition, among anti-CCP+ eRA subjects, a significantly higher titre of anti-CCP antibodies was observed in those presenting with erosions (2993, 1628–3200 IU/ml) versus patients without basal erosions (199, 164–596 IU/ml) (Fig. 4D).

No differences in circulating Th17 or Th17/Th1 cells were observed between RF(+) and RF(-) eRA or established RA patients. Also, no significant correlations were observed between the frequency of circulating Th17 cells and the titre of RF, DAS 28 score, age, ESR, or CRP, in eRA or established RA patients.

### In vivo Effect of Treatment on the Circulating Th17 and Th17/Th1 Frequencies

**Figure 5:**
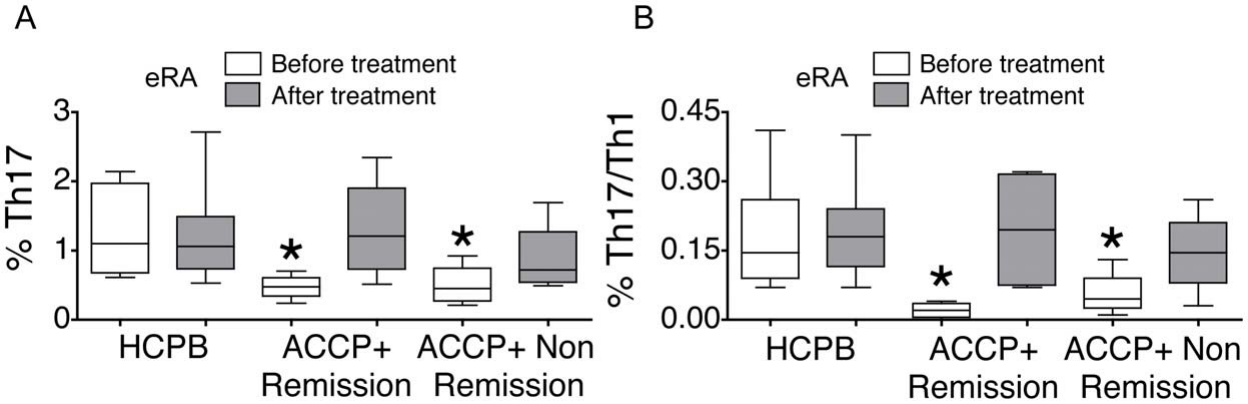
In vivo effect of treatment on the circulating Th17 and Th17/Th1 frequencies. Eight patients with anti-CCP+ eRA donated blood for a second time, one year after the first visit, and while receiving treatment with oral MTX with or without low-dose prednisone. Four out of these patients had achieved remission and 4 of them demonstrated persistent disease activity. Shown are the frequencies of circulating Th17 and Th17/Th1 cells in patients who achieved remission (ACCP+ remission) (n = 4), in patients who did not achieve remission (ACCP-non-remission) (n = 4) and in their age and sex-matched controls (HCPB) (n = 8), observed at first evaluation (“before treatment”) and at the one-year follow-up visit (“after treatment”). Box plots represent the median and interquartile range of all studied subjects, whiskers represent the maximum and minimum values. *p<0.05 vs HCPB.

Interestingly, when re-evaluated after one year and when patients were receiving treatment with oral MTX with or without low-dose prednisone, the frequencies of circulating Th17 and of circulating Th17/Th1 cells in anti-CCP+ eRA subjects were no longer different from controls (Fig. 5A, B), and this was observed not only in patients who had achieved remission but also in patients who had persistent disease activity. Experimental variation in healthy controls was minimal when comparing data obtained in the first versus the second visit (Fig. 5A, B).

## Discussion

Several previous studies indicate that Th17 cells may play an important role in the pathogenesis of RA [1]–[13]; therefore we hypothesized that their numbers might be augmented in eRA patients. We surprisingly detected a significantly decreased frequency of circulating Th17 cells when analyzing all of our patients with eRA as a single group, whereas established RA patients showed circulating Th17 frequencies that were not different from controls. Remarkably, after dividing eRA patients according to the presence or absence of anti-CCP antibodies, it was evident that only anti-CCP positive eRA patients demonstrated a decreased circulating Th17 population; in contrast, the frequency of circulating Th17 cells in anti-CCP negative eRA was comparable to the one observed in healthy controls. That is, despite the fact that anti-CCP+ patients represent 45% of our eRA population sample, their markedly decreased Th17 frequency is able to bring down the final numbers in the total eRA group, low enough to result in a significant difference in comparison with healthy controls. At the same time, the frequency of total peripheral blood CD4+ T cells, the frequency of circulating Th1 cells, and the secretion of CD4-derived IFN-γ, TNF-α and IL-10, were not different between eRA patients and healthy controls. Of note, the frequency of circulating Th17/Th1 cells was decreased in the peripheral blood of anti-CCP+ but not anti-CCP- eRA patients, consistent with recent observations indicating that IL-17+/IFN-γ+ double producers arise from Th17 and not from Th1 cells [32].

Interestingly, among anti-CCP+ eRA patients, both the Th17 and the Th17/Th1 frequency were negatively correlated with the titre of anti-CCP antibodies which further reinforces the link between low Th17 counts and CCP seropositivity. In addition, a significantly lower frequency of circulating Th17 and Th17/Th1 cells was observed in patients who presented with erosive versus patients presenting with non-erosive eRA; however, after adjusting for anti-CCP antibody status it was evident that this was linked to the strong relation between anti-CCP antibodies and erosions [31].

We chose to analyze cells from the synovial fluid of RA as representative cells from the RA inflammatory site: RA synovial fluid T lymphocytes represent T cells that have reached the joint through the peripheral blood, and have acquired an activated phenotype by locally interacting with the inflamed synovial tissue, where hyperplastic synovial fibroblasts and activated synovial macrophages are abundant [33], [34]. We observed that, when compared with the peripheral blood of healthy subjects and of patients with early or established RA, an increased frequency of Th17 cells was present in RA synovial fluid, together with an increased frequency of Th17/Th1 cells. This is consistent with a set of previous studies on RASF Th17 frequencies [19], [24] but discordant with others [22]. That is, Th17 cells seem to be concentrated in the RA inflammatory focus but spreading of potentially pathogenic Th17 cells through the peripheral blood is limited or decreased, which could be related to increased migration followed by sequestration in the inflamed joint [35]. This situation may be reversed following treatment; in fact, it has been reported that anti-TNF therapy in RA is associated with an increased percentage of circulating Th17 cells possibly attributable to a decreased homing to the synovium [18].

We were particularly interested in examining IL-17 production by other cell populations, such as γδ T cells, CD8 T cells and NK cells, but observed that this cytokine was exclusively produced by CD4+ T cells in RA synovial fluid and in the peripheral blood of healthy controls, eRA and established RA patients, consistent with work published by Shen et al [29].

Th17 biology is complex and incompletely understood, which may explain why previous reports on the frequency of Th17 cells in RA have yielded conflicting results. Some authors have found increased circulating Th17 frequencies [20], [21], [24], [25] whereas others report comparable frequencies to those displayed by healthy controls [18], [19], [22]. Discordances among studies may be due in part to differences in the clinical profile and/or medications of patients. Whereas an influence of immunomodulating drugs on the ex vivo behavior of isolated cells is possible [18], the effect of perpetuating inflammatory feedback loops that are effective as disease progresses may also be a player.

Our population of subjects with early disease and who had never received corticosteroids or DMARDs was not homogeneous when considering the frequency of circulating Th17 cells. A clear difference between anti-CCP+ and anti-CCP- eRA patients was observed, and a ready explanation for this observation is not apparent at present. In fact, the data reported herein may contribute to reinforce the notion that anti-CCP+ and anti-CCP- RA are distinct entities [36].

The different circulating Th17 and Th17/Th1 cell frequencies in patients with early versus established RA suggests that the immune mechanisms mediating early phases of the disease may be different from those implicated in more advanced stages [37]. Interestingly, when our anti-CCP+ eRA patients were re-examined one year after the first visit, while receiving treatment with oral MTX, their circulating Th17 frequency was no longer different from controls. This was apparent not only in patients who had achieved remission but also in patients who had persistent disease activity, which suggests that medication may be playing a role. Other investigators have concluded that the peripheral blood Th17 frequency in RA may be modified by therapy. As noted above, Aerts et al [18] reported that in established RA the peripheral Th17 cell frequency is not elevated, but anti-TNF therapy induces a striking increase of circulating Th17 cells and IL-17 production, irrespective of disease activity. In addition, Notley et al [38] found that in collagen-induced arthritis, TNF blockade resulted in reduced arthritis severity but, unexpectedly, expanded populations of extra-articular Th17 cells, which were shown by adoptive transfer to be pathogenic.

Finally, recently published phase II clinical trials on the efficacy of IL-17 blockade in RA have yielded conflicting results [39], [40]. In light of our findings, discrepancies could be related to differences in the stage of disease or anti-CCP antibody status of patients.

### Conclusion

In summary, we have observed a significantly decreased frequency of circulating Th17 and Th17/Th1 cells in anti-CCP+ eRA patients that returns to values observed in controls during follow-up after initiating treatment with MTX. The basal circulating Th17 frequency was significantly correlated with the titre of anti-CCP antibodies and with the presence or absence of basal erosions. At the same time, the basal Th17 and Th17/Th1 frequency in anti-CCP- eRA patients was not different from controls. This indicates that further studies are needed to fully understand the complex biology of Th17 cells in RA. In addition, the percentage of circulating Th17 cells in eRA can be considered as a marker of CCP positivity.
